# Supplementary material for: Fluorescence Spectroscopy of Low-Level Endogenous β-Adrenergic Receptor Expression at the Plasma Membrane of Differentiating Human iPSC-Derived Cardiomyocytes
Source: Int J Mol Sci. 2022 Sep 8;23(18):10405. doi: 10.3390/ijms231810405 (PMC9499492; doi:10.3390/ijms231810405)
Supplement: Supplementary file 1 [file ijms-23-10405-s001.zip › 220909 Supplementary Materials_3rd revised MS_tracked changes.pdf]

Supplementary Materials

Fluorescence Spectroscopy of Low-Level Endogenous β-adrenergic Receptor Expression at the Plasma Membrane of Differentiating Human iPSC-Derived Cardiomyocytes

Philipp Gmach[1,2,†], Marc Bathe-Peters[1,†], Narasimha Telugu[1], Duncan C. Miller[1,3] and Paolo Annibale[1,3,4,*]

1 Max Delbrück Center for Molecular Medicine in the Helmholtz Association, 13125 Berlin, Germany
2 Institute of Pharmacology and Toxicology, University of Würzburg, 97070 Würzburg, Germany
3 German Centre for Cardiovascular Research, partner site Berlin, 10785 Berlin, Germany
4 School of Physics and Astronomy, University of St Andrews, St Andrews KY16 9AJ, UK
* Correspondence: pa53@st-andrews.ac.uk
† These authors contributed equally to this work.

Content

Figure S1. Timeline displaying differentiation and maturation of hiPSC-CMs 2

Figure S2. Strategy for generation and identification of the endogenously CRISPRed *HA-mEGFP-ADRB2* clones 3

Figure S3. Pluripotency characterization of WT hiPSCs and the CRISPR clones 4

Figure S4. (A) Mean diffusion constants and (B) mean number of particles extracted from the autocorrelation curves in Figure 3 5

Figure S5. HA-mEGFP-ADRB2 overexpression in H9c2 cells 6

Video S1. Spontaneous contractions of the hiPSC-CM clone 16-31 (at day 100) before β_2-AR stimulation and after 25 min of 300 nM CGP-20712A addition 7

Video S2. Spontaneous contractions of the hiPSC-CM clone 16-31 (at day 100) after 5 min of β_2-AR stimulation 7

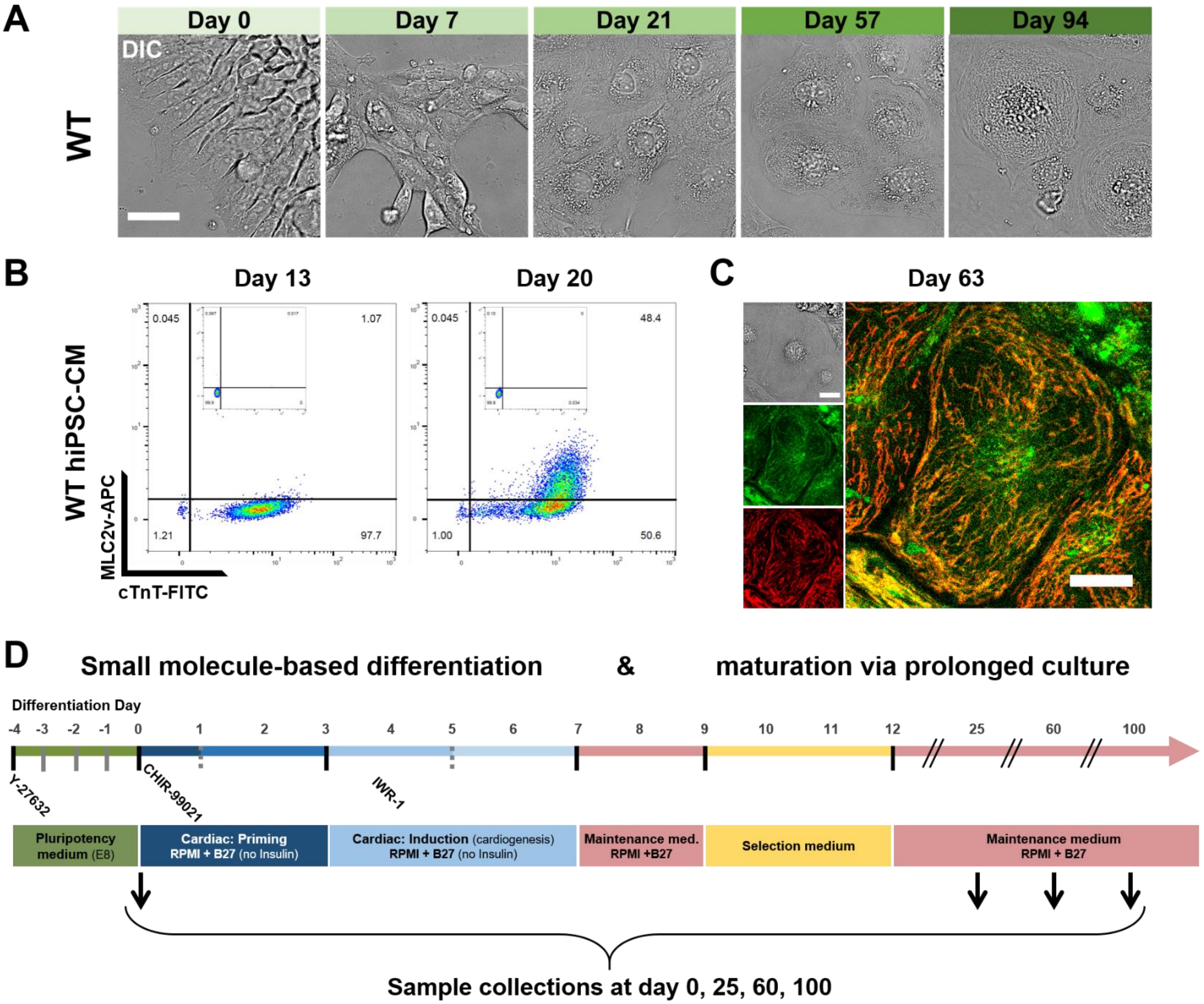

Figure S1. Timeline displaying differentiation and maturation of hiPSC-CMs. (A) Shows changes of cellular morphology of undifferentiated WT hiPSC (day 0) and WT cells differentiated and matured in 2D monolayers for 7, 21, 57 and 94 days in culture. Scale bar is 30 µm. (B) Representative flow cytometry plots indicating the expression of cardiac-specific markers (cardiac Troponin T (cTnT) and the ventricular isoform of the myosin light chain 2 (MLC2v)) at day 13 and day 20 of differentiation of the parental WT hiPSC-CM line (BIHi005-A), differentiated according to the protocol described in the methods, are shown. Insets display isotype control staining. (C) Mitochondrial networks of day 63 WT hiPSC-CMs superposed to autofluorescence signal. Counterclockwise from top left: DIC (gray), autofluorescence at 488nm (green), 50 nM MitoTracker Deep Red FM stain (red) and overlay of autofluorescence together with MitoTracker. Scale bars are 20 µm. (D) Differentiation strategy based on small molecules (see Methods) depicting the most important treatment media indicated by day and duration of treatment and days of sample collection (25 +/-4 days; 60 +/-4 days; 100 +/-7 days).

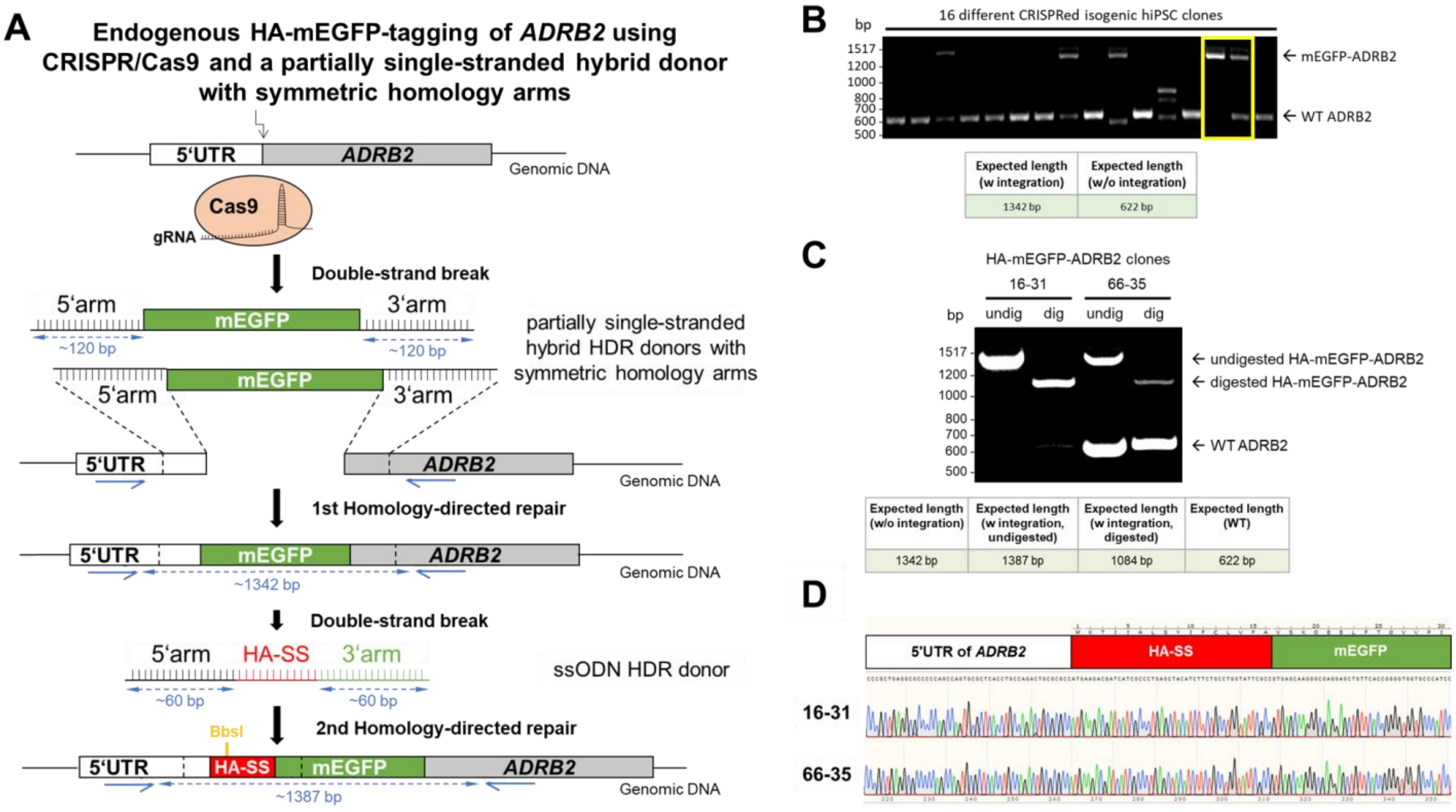

Figure S2. Strategy for generation and identification of the endogenously CRISPRed *HA-mEGFP-ADRB2* clones. (A) Schematic of the 2-step CRISPR approach for N-terminal mEGFP-tagging of *ADRB2* based on partially single-stranded hybrid donors with symmetric homology arms for the 1st homology-directed repair (HDR). In a 2nd CRISPR step the influenza hemagglutinin signal sequence (HA-SS) (MKTIIALSYIFCLVFA) was introduced endogenously at the N-terminus of *mEGFP-ADRB2* for improved membrane targeting using a single-stranded oligodeoxynucleotide (ssODN) as HDR donor. (B) PCR genotyping of 16 CRISPRed isogenic hiPSC clones on agarose gel after 1st homology-directed repair based on genomic PCR using primers that bind outside the homology arms of the HDR donor as indicated with blue arrows in (A). Homozygous (clone 16) and heterozygous (clone 66) clones are highlighted in the yellow rectangle. (C) Genotyping of two isogenic *HA-mEGFP-ADRB2* CRISPRed clones 16-31 and 66-35 which were derived from *mEGFP-ADRB2* CRISPRed clones 16 and clone 66 (highlighted in (B)) after using them for the 2nd CRISPR step (introducing HA-SS). Clones were identified by restriction fragment length polymorphism (RFLP) using the BbsI restriction enzyme cut site in the HA-SS (highlighted in orange in A). Shown are undigested (undig) and BbsI-digested (dig) genomic PCR reactions and the expected resulting PCR fragment sizes. (D) Shows DNA chromatograms of clones 16-31 and 66-35 based on Sanger sequencing at their CRISPRed *ADRB2* loci verifying correct in frame integration of the HA-mEGFP sequence at the N-terminus of *ADRB2*.

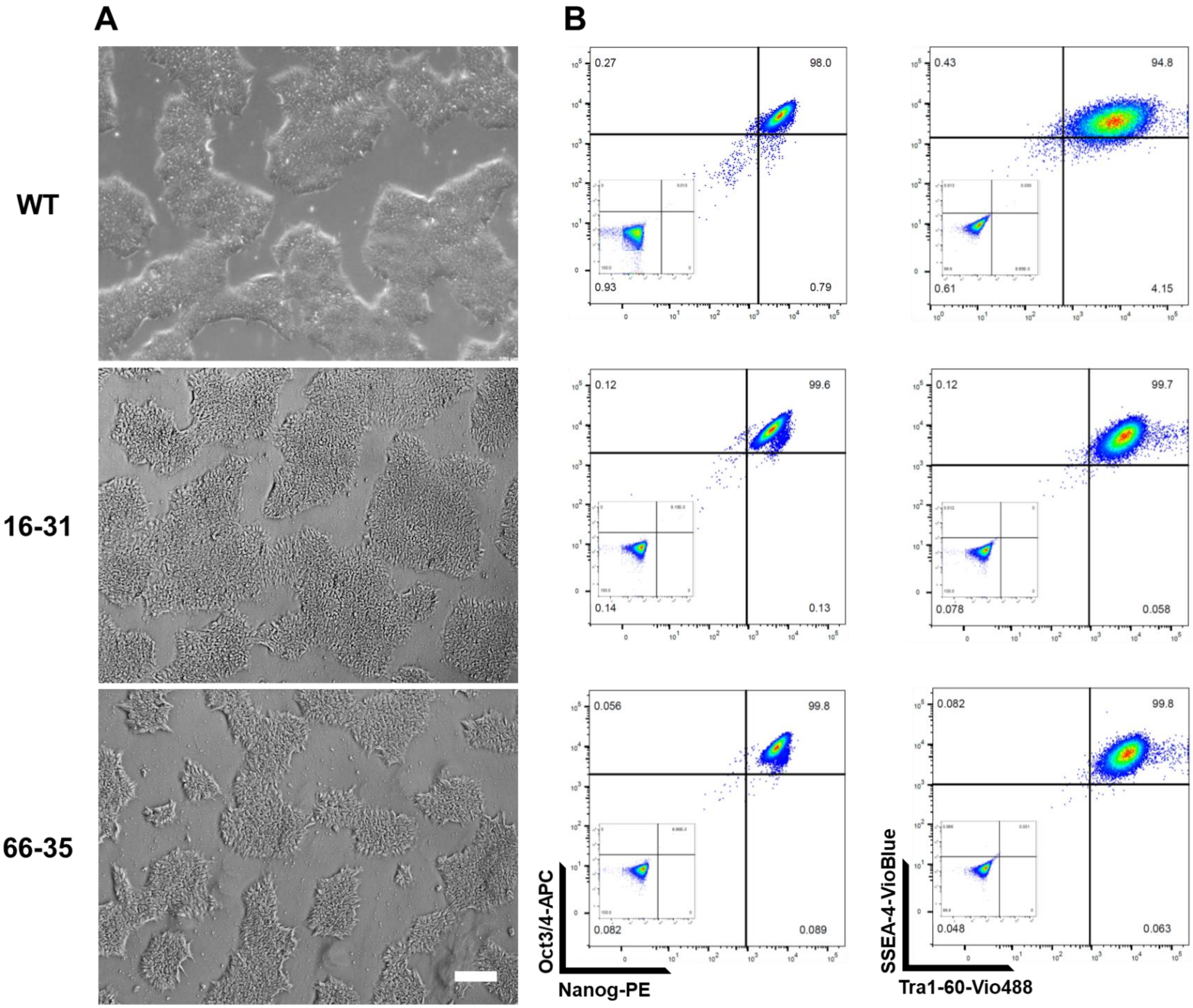

Figure S3. Pluripotency characterization of WT hiPSCs and the CRISPR clones. (A) Brightfield images indicate stem cell typical growth in colonies for undifferentiated WT hiPSC and both clones 16-31 and 66-35. Scale is 250 µm. (B) Representative flow cytometry plots indicating expression of the 4 pluripotency marker genes Oct3/4 and Nanog (left column) as well as SSEA-4 and Tra1-60 (right column) in all three cell lines used. Insets display isotype control staining.

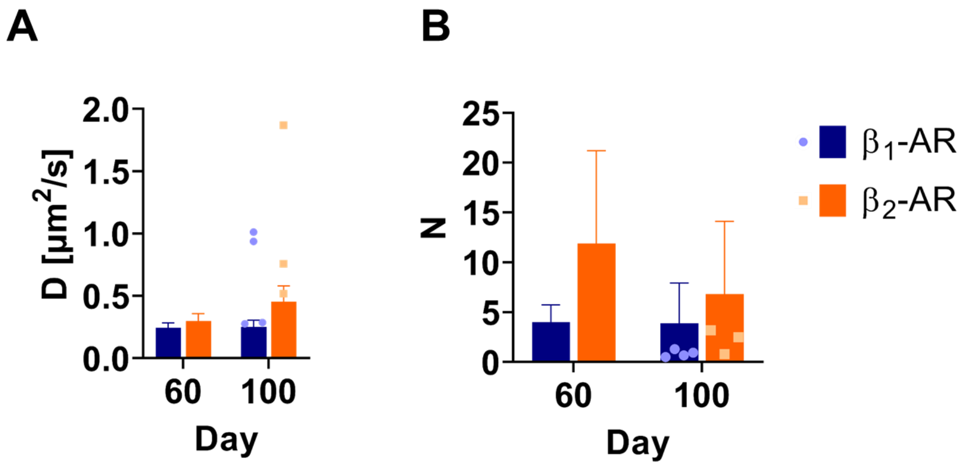

Figure S4. (A) Mean diffusion constants and (B) mean number of particles extracted from the autocorrelation curves in Figure 3. Error bars represent SD values. Dots and small squares indicate diffusion constants and receptor numbers extracted at day 107 (and are not part of the day 100 mean).

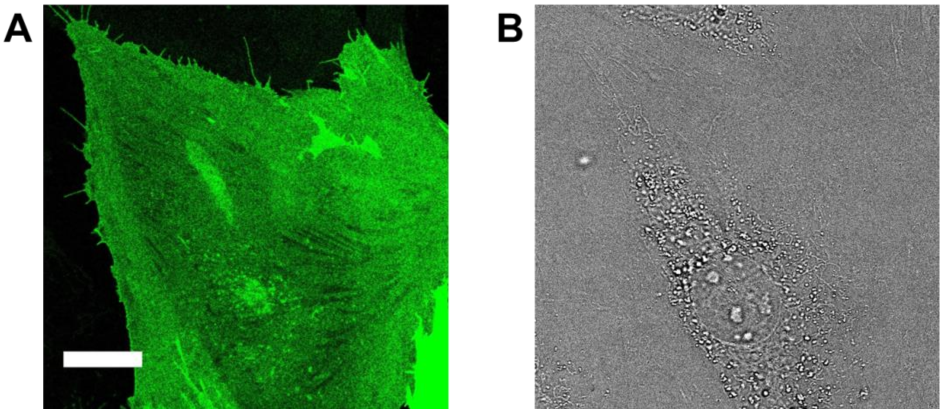

Figure S5. HA-mEGFP-ADRB2 overexpression in H9c2 cells. (A) Confocal micrograph of the basolateral membrane of an H9c2 cell expressing the construct HA-mEGFP-ADRB2, excited at 488 nm. (B) corresponding DIC image. Scale bar is 20 μm.

Video S1. Spontaneous contractions of the hiPSC-CM clone 16-31 (at day 100) before β_2-AR stimulation and after 25 min of 300 nM CGP-20712A addition.

Video S2. Spontaneous contractions of the hiPSC-CM clone 16-31 (at day 100) after 5 min of β_2-AR stimulation.
